# Assessing Two Dimensions of Interpersonal Trust: Other-Focused Trust and Propensity to Trust

**DOI:** 10.3389/fpsyg.2021.654735

**Published:** 2021-07-27

**Authors:** Ming Zhang

**Affiliations:** ^1^CAS Key Laboratory of Behavioral Science, Institute of Psychology, Chinese Academy of Sciences, Beijing, China; ^2^Department of Psychology, University of Exeter, Exeter, United Kingdom

**Keywords:** other-focused trust, propensity to trust, scale development, interpersonal interaction, social interaction

## Abstract

One’s propensity to trust others and others’ trustworthiness are two important aspects of interpersonal trust. Both theory and research suggest that it is possible to distinguish between an individual’s propensity to trust (one’s “trustingness” or the extent to which one feels able to trust others) and their other-focused trust (the extent to which one feels that others are worthy of our trust). However, there is as yet no measure that distinguishes between these two components of trust. In three studies, we examined the psychometrics of a proposed two-dimensional measure of trust that encompasses propensity to trust and other-focused trust components. To test discriminant validity, we also administered measures of personality, personal self-esteem, social capital, propensity to like people, perceived social support, as well as general and personal beliefs in a just world. Factor analyses supported the proposed two-factor model for the new trust measure. Further analyses supported the difference between these measures.

## Introduction

Trust is commonly defined as a confident expectation about a situation leading to a willingness to accept vulnerabilities that arise from uncertainty and risk (Dietz, 2011; Patent and Searle, 2019). Trust plays an important role in daily interpersonal interactions (Rotenberg et al., 2005), predicting individual behavior toward others (Colquitt and Salam, 2009; Yamagishi et al., 2015). Trust is also directly and strongly linked to individual well-being, health, and longevity (Young and McGrath, 2021).

Trust involves a willingness to be vulnerable to the actions of another person or people (Mayer et al., 1995; McEvily and Tortoriello, 2011). This implies that when trusting others, individuals often simultaneously bring together their own propensity to trust other people with their judgment of whether specific others deserve their trust (Colquitt et al., 2007; Lucas et al., 2011). Considering interpersonal interaction included the individual’s side and the other person’s side (Dietz and Den Hartog, 2006; Schoorman et al., 2007; Jones and Shah, 2016), it means the interpersonal trust depended on both two parties. Thus, interpersonal trust should be considered from two aspects: the general trustworthiness of others (i.e., a state-based trust, depends on other people) and one’s own general propensity to trust others (i.e., a trait-based trust, depends on oneself). There were series of studies focused on trustworthiness, propensity to trust, or both of them (Mayer and Davis, 1999; Frazier et al., 2013; Alarcon et al., 2018a,b; Patent and Searle, 2019). However, these two specific aspects of trust have not been clearly conceptualized or empirically differentiated by the items with clear face validity that described trust from the viewpoint of others and oneself (“people” vs. “I”).

The most common definitions of trust refer to the extent to which we think others are trustworthy (Mayer et al., 1995)—this is what we refer to as “other-focused trust.” This refers to the state-based general trustworthiness of others, that is, the judgment individuals make about whether or not the other(s) is (are) deserving of their trust, for example as a function of their perceived integrity and good character (Colquitt and Rodell, 2011). Individuals arrive at this judgment based on their observation of, or inference about, other people’s behaviors, or based on pre-existing (and often prejudiced) beliefs about them (Evans and Revelle, 2008). The extent to which we find specific others trustworthy can vary depending on who others are and our experiences with and beliefs about them, but people can also have more chronic tendencies to believe that particular others are worthy of trust, based for example on past experiences where trust was confirmed, or instead broken (Murray, 2015).

In addition to focusing on whether others are trustworthy, individuals can be (or feel) more or less trusting, or inclined to trust other people (Burke et al., 2007). For example, individuals who have undergone traumatic experiences might temporarily withdraw trust in others, irrespective of who they are (Jansen et al., 2015). That is, they might feel they cannot trust other people because of how they are feeling, rather than because of who the other is—this is what we designate here as “propensity to trust.” As defined here, propensity to trust is self-focused, less likely to vary depending on who others are and more likely to respond to variations in the individuals’ sense of safety and esteem (Mayer et al., 2009; Beldad et al., 2010). When high, propensity to trust reflects an optimistic conception of the world and generalized positive expectations about others (Zeffane, 2020). This also highlights that propensity to trust and other-focused trust—as two dimensions of interpersonal trust—are likely to be closely related, though possible to differentiate.

Two-dimensional models of trust have been proposed before (see Thielmann and Hilbig, 2015 for a review). The closest model to what we are proposing in this paper is by Yamagishi et al. (2015). These authors introduced a two-component trust scale that differentiates between “trust beliefs” and “trust preferences.” Trust beliefs refer to an individual’s estimate about the trustworthiness of others—a sample item is “Most people are trustworthy.” Though this subscale includes items that do not quite match our conceptualization of other-focused trust (e.g., “Generally, I trust others”), this is similar to what is assessed by most traditional measures of trust (e.g., Yamagishi and Yamagishi, 1994) and to what we designate here as “other-focused trust.” Trust preferences, by contrast, refer to an individual’s propensity to be vulnerable to others and reflect a particular sense of self as trusting in which “people derive personal satisfaction from being a trustful person.” Trust preference, by contrast, refers to an individual’s propensity to be vulnerable to others and reflects a particular sense of self (Yamagishi et al., 2015, p. 440).

Though this conceptualization by Yamagishi et al. (2015) is similar to what we propose here for “willingness to trust,” it differs from ours by explicitly incorporating a willingness to incur costs as a function of trusting others (e.g., “Even though I may sometimes suffer the consequences of trusting someone, I still prefer to trust than not to trust others”). Though this choice is likely to increase the empirical distinction between the two subscales, it ignored one situation. Specifically, there was a limitation in revealing as trusting those who are unlikely to habitually consider the consequences of their trust, but who if asked in this way might feel foolish to admit that they would be trusting if their trust were to be abused. This is also important because self-report measures are notorious for their vulnerability to impression management (Chan, 2009), and portraying oneself as open to abuse is not generally seen as desirable. As such, our aim was to develop a similar measure to that of Yamagishi et al. (2015), but which did not refer to the costs people are willing to incur for being trusting. This measure would allow researchers to, in the future, shed further light on the mechanisms underlying trust, or its consequences, by examining their differential drivers, how they might vary across contexts, and how they differentially guide behavior in social interactions.

As part of this development, we also aimed to examine whether propensity to trust and other-focused trust were differently associated with measures that have been previously shown to be related to trust. Previous research has shown that trust is associated with a range of personality traits (De Jong et al., 2016), as assessed by the Big Five Personality Inventory (Evans and Revelle, 2008), life satisfaction (Abbott et al., 2016), and personal self-esteem (Bao et al., 2016). For instance, perceived trustworthiness (other-focused trust) has been shown to be positively correlated with agreeableness and with conscientiousness (Evans and Revelle, 2008). People’s propensity to like others has also been shown to be positively related to trust, especially in the relationship between buyers and sellers (Nicholson et al., 2001). Trust is also closely related to social capital (Son and Feng, 2019) and perceived social support (Shin, 2013). For example, communities with higher levels of social capital tend to include citizens who are more trusting of one another and those who report receiving social support from others usually have more generous expectations of others, including greater trust (Ikeda, 2013). Trust is also likely to be related to an individual’s beliefs in a just world. The belief in a just world is defined as the perception that one lives in a world where people generally get what they deserve (Trost et al., 2014). People want to believe they live in a just world so that they can go about their daily lives with a sense of trust, hope, and confidence in their future so a high level of belief in a just world is likely to be related to a high level of trust (Schindler and Reinhard, 2015).

Although prior research has examined the relationship between trust and these other constructs, and distinguished different components of trust, there has been as yet no attempt to distinguish the correlates of propensity to trust and other-focused trust. Our goal is to develop a scale that allows for their separate measurement and to examine the differential associations between these two types of trust and personality variables, personal self-esteem, life satisfaction, social capital, social support, and belief in a just world.

## Study 1: Item Selection

The key goal of this study was to develop items that have the face and construct validity as measures of propensity to trust and other-focused trust. First we selected a pool of items from existing scales based on their face validity regarding the two hypothesized components. Second, we conducted exploratory factor analyses to investigate whether the items could be divided into two sets, assessing propensity to trust and other-focused trust. Third, we explored the associations between the two subscales and other relevant variables previously shown to be associated with aspects of trust.

### Materials and Methods

#### Participants

Participants were recruited through the online research crowdsourcing platform Prolific Academic. After excluding 11 participants who failed the attention check, a total of 491 adults (age: 35.12 ± 11.54 years) participated in this study, with a participants-to-variables ratio of 18:1, based on the criterion established by Worthington and Whittaker (2006). Table 1 reports the demographic information, education level, and nationality of participants. Due to the available distribution of gender and education, we examined the effect of gender and education on trust scores for all three studies (see Supplementary Material).

**TABLE 1:** Participant characteristics.

	**Study 1 (*N* = 491)**		**Study 2 (*N* = 501)**		**Study 3 (*N* = 295)**	
	***n***	**%**	***n***	**%**	***n***	**%**
**Gender**						
Male	197	40.1	155	30.9	75	25.4
Female	290	59.1	343	68.5	220	74.6
Other	3	0.6	3	0.6	0	0
Missing	1	0.2	0	0	0	0
**Nationality**						
British	430	87.6	446	89.0	261	88.5
Non-British European	38	7.7	46	9.2	24	8.1
North American	10	2.0	0	0	1	0.3
Non-European and non-North American	13	2.7	9	1.8	9	3.1
**Education**						
High school diploma or equivalent	165	33.6	234	46.7	123	41.7
Bachelor’s degree or equivalent	231	47.0	185	36.9	109	36.9
Master’s degree or equivalent	69	14.1	48	9.6	51	17.3
Ph.D. or equivalent	12	2.4	20	4.0	4	1.4
Other	14	2.9	14	2.8	8	2.7

#### Measures

An initial pool of 27 items was selected from three trust scales: Three items used in a study by Baltatescu (2009), 19 items from the Generalized Trust Scale (Couch et al., 1996), and 5 items from the General Trust Scale (Yamagishi and Yamagishi, 1994). The full wording of all items is provided in Table 2. All items were selected based on face validity regarding their potential to tap into the aforementioned definitions of propensity to trust and other-focused trust. Participants responded to each item on a Likert-type scale from 1 = *strongly disagree* to 5 = *strongly agree*. In order to explore possible differences between the two dimensions of interpersonal trust, participants also completed four individual difference measures including the Big Five Inventory (e.g., “I see myself as someone who is talkative”; John and Srivastava, 1999), the Liking People Scale (e.g., “Sometimes when people are talking to me, I find myself wishing that they would leave”; Filsinger, 1981), Rosenberg’s Personal Self-Esteem Scale (e.g., “On the whole, I am satisfied with myself”; Rosenberg, 1965), and the Satisfaction with Life Scale (e.g., “In most ways my life is close to my ideal”; Diener et al., 1985).

**TABLE 2:** All 27 items used for testing in Study 1.

**Items**	**Scale**
1. Most people can be trusted.	Baltatescu, 2009
2. Most people try to take advantage of me.	
3. Most of the time people are helpful.	
4. I tend to be accepting of others.	Couch et al., 1996
5. My relationships with others are characterized by trust and acceptance.	
6. Basically I am a trusting person.	
7. It is better to trust people until they prove otherwise than to be suspicious of others until they prove otherwise.	
8. I accept others at “face value.”	
9. Most people are trustworthy.	
10 It is better to be suspicious of people you have just met, until you know them better.	
11. I make friends easily.	
12. Only a fool would trust most people.	
13. I would admit to being more than a little paranoid about people I meet.	
14. I have few difficulties trusting people.	
15. Basically, I tend to be distrustful of others.	
16. Experience has taught me to be doubtful of others until I know they can be trusted.	
17. I have a lot of faith in the people I know.	
18. Even during “bad times,” I tend to think that things will work out in the end.	
19. I tend to take others at their word.	
20. When it comes to people I know, I am believing and accepting.	
21. I feel I can depend on most people I know.	
22. I almost always believe what people tell me.	
23. Most people are basically honest.	Yamagishi and Yamagishi, 1994
24. Most people are basically good and kind.	
25. Most people are trustful of others.	
26. I am trustful.	
27. Most people will respond in kind when they are trusted by others.	

#### Procedure

The study was programmed using Qualtrics and distributed on the Prolific Academic crowdsourcing platform. The survey protocol was approved by the University of Exeter’s Psychology Ethics Committee. Participants were invited to participate in a study on trust and offered £1 in compensation, in line with compensation standards on this platform. After providing informed consent, participants provided basic demographic information, including gender, age, nationality, and education. Next, participants responded to the total pool of 27 items assessing interpersonal trust. Finally, participants responded to the 44-item Big Five Inventory, the 15-item Liking People Scale, the 10-item Personal Self-Esteem Scale, and the 5-item Satisfaction with Life Scale.

### Results

#### Exploratory Factor Analyses

Principal components analysis was used to identify the items that would best distinguish between propensity to trust and other-focused trust. Oblimin rotation was used to interpret the factor loadings because propensity to trust and other-focused trust were expected to be correlated. The Kaiser-Meyer-Olkin measure of sampling adequacy (KMO = 0.94) and Bartlett’s test of sphericity, χ^2^(325) = 6470.04, *p* < 0.001, showed adequate fit. It was observed that 26 of the 27 items correlated at least 0.30 with at least one other item, suggesting reasonable factorability. The communalities for the 26 items were all above 0.30, further confirming that each item shared some common variance with other items. Given these overall indicators, factor analysis was deemed to be suitable.

Initial eigenvalues (see Table 3) in principal components analysis indicated that the first five factors explained 38.65, 7.17, 5.57, 4.66, and 4.24% of the variance in the items. Rotation sums of squared loadings indicated that the first five factors explained 6.24, 4.36, 7.28, 4.80, and 3.93% of the variance. Two criteria were used to determine the factor structure: (a) Retain items with a factor loading equal to or greater than 0.30 and (b) Items with double loadings were carefully considered and retained only where their content matched that of other items within a factor on which they loaded sufficiently. The first factor consisted merely of all reversed items and was therefore not theoretically meaningful. The second and fourth factors had a very low number of primary loadings. However, the third and fifth factors had a sufficient number of primary loadings that were theoretically meaningful and distinguished between the two theorized components. Therefore, the 12 items loading on these two factors were retained.

**TABLE 3:** Factor loadings and communalities for the 27 items in Study 1.

**Items**	**Factors loading**					**Communality**
	**Component 1**	**Component 2**	**Component 3**	**Component 4**	**Component 5**	
13. I would admit to being more than a little paranoid about people I meet.	–0.717					0.565
10 It is better to be suspicious of people you have just met, until you know them better.	–0.713					0.649
16. Experience has taught me to be doubtful of others until I know they can be trusted.	–0.683					0.727
15. Basically, I tend to be distrustful of others.	–0.627					0.722
12. Only a fool would trust most people.	–0.625					0.666
2. Most people try to take advantage of me.	–0.564					0.559
7. It is better to trust people until they prove otherwise than to be suspicious of others until they prove otherwise.	0.445					0.544
22. I almost always believe what people tell me.		0.720				0.608
19. I tend to take others at their word.		0.616				0.639
8. I accept others at “face value.”		0.600				0.527
24. Most people are basically good and kind.			0.801			0.757
3. Most of the time people are helpful.			0.777			0.585
23. Most people are basically honest.			0.770			0.702
9. Most people are trustworthy.			0.744			0.777
1. Most people can be trusted.			0.716			0.745
27. Most people will respond in kind when they are trusted by others.			0.639			0.519
17. I have a lot of faith in the people I know.				–0.858		0.745
21. I feel I can depend on most people I know.				–0.797		0.675
20. When it comes to people I know, I am believing and accepting.				–0.726		0.691
11. I make friends easily.					–0.792	0.564
4. I tend to be accepting of others.					–0.518	0.484
6. Basically I am a trusting person.		0.471			–0.477	0.696
26. I am trustful.					–0.459	0.565
18. Even during “bad times,” I tend to think that things will work out in the end.					–0.433	0.309
5. My relationships with others are characterized by trust and acceptance.				–0.400	–0.424	0.475

A principal components factor analysis of the remaining 12 items was conducted using oblimin rotations. All items in this analysis had primary loadings on one factor over 0.50 except one item (item 18). After excluding this item, a principal components factor analysis of the remaining 11 items using oblimin rotations was conducted again. All items in this analysis had primary loadings over 0.50 on one of the two factors obtained. The factor loading matrix for this final solution is presented in Table 4. These 11 items were retained.

**TABLE 4:** Factor loadings for the items in Study 1 and Study 2.

**Factor items**	**Study 1**			**Study 2**	
	***M (SD)***	**Factor loadings**	**Communality**	***M (SD)***	**Standardized factor loading**
**Other-focused trust**					
1. Most people are trustworthy.	3.49 (1.05)	0.908	0.793	3.31 (1.00)	0.807
2. Most people are basically good and kind.	3.68 (0.94)	0.841	0.750	3.66 (0.94)	0.792
3. Most people are basically honest.	3.44 (1.01)	0.870	0.713	3.36 (1.00)	0.778
4. Most people can be trusted.	3.40 (1.11)	0.895	0.760	3.37 (0.99)	0.853
5. Most of the time people are helpful.	3.79 (0.82)	0.672	0.492	3.79 (0.85)	0.667
6. Most people will respond in kind when they are trusted by others.	4.05 (0.80)	0.558	0.434	–	–
**Propensity to trust**					
1. Basically I am a trusting person.	3.75 (1.12)	0.697	0.649	3.92 (1.07)	0.763
2. I am trustful.	3.91 (1.00)	0.691	0.605	4.22 (0.92)	0.699
3. I make friends easily.	3.07 (1.25)	0.688	0.405	3.35 (1.23)	0.480
4. I tend to be accepting of others.	4.01 (0.87)	0.642	0.515	3.98 (0.88)	0.633
5. My relationships with others are characterized by trust and acceptance.	4.07 (0.84)	0.679	0.443	4.06 (0.78)	0.537

#### Internal Consistency

Internal consistency for each of the scales was examined using Cronbach’s alpha. Both subscales demonstrated good reliability, with *a* = 0.89 for the other-focused trust sub-scale (6 items) and *a* = 0.75 for the propensity to trust sub-scale (5 items). The average inter-item correlations, which were used in predictor and criterion constructs (Diamantopoulos et al., 2012), were *r* = 0.58 for the other-focused trust items and *r* = 0.38 for the propensity to trust items.

#### Associations With Criterion Measures

To examine convergent and discriminant validity, correlations between the two components of interpersonal trust and the individual difference variables were computed (see Table 5). Other-focused trust was moderately and positively correlated with agreeableness (*r* = 0.41, *p* < 0.001). Propensity to trust was highly positively correlated with agreeableness (*r* = 0.63, *p* < 0.001), and moderately positively correlated with extraversion (*r* = 0.44, *p* < 0.001). Other-focused trust and propensity to trust were both negatively associated with neuroticism (*r* = –0.27, *p* < 0.001; *r* = –0.30, *p* < 0.001, respectively) and liking people (*r* = –0.34, *p* < 0.001; *r* = –0.47, *p* < 0.001, respectively). Other-focused trust was positively correlated with propensity to trust (*r* = 0.50). We compared the correlations between the two types of trust and the criterion measures to check whether they were significantly different from each other (Table 5), by z-score for the difference between two correlations in the online calculator^1^.

**TABLE 5:** Comparison of correlation coefficients in Study 1.

	**Correlation coefficients**		***Z*-Score**	***p***
	**Other-focused trust**	**Propensity to trust**		
Extraversion	0.132***	0.436***	–5.23	**<0.001**
Agreeableness	0.411***	0.627***	–4.68	**<0.001**
Conscientiousness	0.153***	0.201***	–0.77	0.439
Neuroticism	–0.274***	–0.300***	0.44	0.658
Openness	0.134***	0.216***	–1.32	0.186
Liking People	–0.341***	–0.465***	2.32	**0.020**
Self esteem	0.233***	0.288***	–0.92	0.357
Life Satisfaction	0.212***	0.269***	–0.95	0.344

***p < 0.001. Bold values represent significant results.

$$z=\frac{\left(\frac{1}{2}\,\text{ln}\,\frac{1+r\,1}{1-r\,1}\right)-\left(\frac{1}{2}\,\text{ln}\,\frac{1+r\,2}{1-r\,2}\right)}{\sqrt{\left(\frac{1}{n\,1-3}+\frac{1}{n\,2-3}\right)}}$$

The results showed that propensity to trust had a significantly stronger correlation with extraversion, agreeableness, and liking people compared to other-focused trust.

### Discussion

The results show that interpersonal trust can be empirically divided into two components: Propensity to trust and other-focused trust. This is consistent with what has already been reported by Yamagishi et al. (2015), but is now shown with subscales that do not also differ in the extent to which the consequences of one’s trust are explicitly considered. The factor analysis supported this distinction in the items we sampled and the resulting subscales proved to be internally consistent. Correlational analysis with other variables showed that propensity to trust and other-focused trust were both positively correlated with some of the measures included in this study (extraversion, agreeableness, conscientiousness, openness, self-esteem, and life satisfaction) and negatively correlated with neuroticism and liking of others. However, by comparing the magnitude of the correlations between the two types of trust and criterion measures, we found evidence that the two types of trust were distinguishable in some aspects. Specifically, propensity to trust was more strongly correlated with extraversion, agreeableness, and liking people than other-focused trust. This suggests that these facets of personality may reflect a broader interpersonal orientation that is more strongly reflected in propensity to trust than other-focused trust (which is less about the self). These divergences support the idea that the measure we developed taps into two forms of trust that can be empirically differentiated.

## Study 2: Confirmatory Analyses

The key goal of Study 2 was to confirm the construct validity of the two subscales. We also aimed to extend the test of the scales’ discriminant validity by adding a social capital scale and a scale of perceived social support. We kept personal self-esteem in this study, to replicate the relationship found in Study 1.

### Materials and Methods

#### Participants

A total of 501 participants (age: 35.96 ± 11.79 years) were recruited through the Prolific Academic platform (see Table 1 for more demographic information).

#### Measures

The 11-item other-focused and propensity to trust scale developed in Study 1 was used. In addition, participants completed the 6-item revised Social Capital Scale (e.g., “People around where I live are willing to help others”; Martin et al., 2004), the 6-item brief form of the Perceived Social Support Questionnaire (e.g., “Where I live, people give others a lot of understanding and security”; Kliem et al., 2015), and the same Personal Self-Esteem scale used in Study 1. We revised these items in Social Capital Scale and Perceived Social Support Questionnaire by adding “where I live” to limit the context for the situation described by these items (please see full wording in the Appendix).

#### Procedure

The study was programmed using Qualtrics and distributed on the Prolific Academic platform. The survey protocol was approved by the University of Exeter’s Psychology Ethics Committee. After providing informed consent, participants provided basic demographic information, including gender, age, nationality, and education. Next, participants completed the items used to assess trust, self-esteem, social capital, and perceived social support, in this order. All participants were compensated with £0.5, as appropriate on this platform.

### Results

#### Confirmatory Factor Analysis

Confirmatory Maximum Likelihood Factor Analysis was used to test the goodness of fit of the two-factor model using AMOS 24.0 for SPSS. The covariance structure was analyzed and the scale was set using the disturbance term of hypothesized latent variables (Lucas et al., 2011). Model fit was evaluated using χ^2^ goodness of fit, the root mean square error of approximation (RMSEA), the non-normed fit index (NFI), and the comparative fit index (CFI). Acceptable fit was indicated by an RMSEA of 0.08 or below, and values above 0.90 for the NFI and CFI (Effendi and Shunhaji, 2020).

The model fit was first obtained for a one-factor model, revealing poor fit: χ^2^(44, *N* = 501) = 404.636, *p* < 0.001, NFI = 0.824, CFI = 0.839, and RMSEA = 0.128 (90% confidence interval: 0.117, 0.140). Then, the model fit was obtained again for the proposed two-factor model, showing relatively good fit: χ^2^(43, *N* = 501) = 188.34, *p* < 0.001, NFI = 0.918, CFI = 0.935, and RMSEA = 0.082 (90% confidence interval: 0.070, 0.094). Item loadings for the two specified factors were significant at *p* < 0.001, and ranged for each dimension as follows: Other-focused trust (0.398–0.851) and propensity to trust (0.479–0.762). The two latent constructs were significantly correlated with one another (*r* = 0.67, *p* < 0.001). In the other-focused trust subscale, there was an item with a factor loading lower than 0.40, so this item was dropped based on the criterion of Moore et al. (2020), which views a coefficient of 0.40 as the minimum level for a variable to contribute meaningfully to a factor.

After dropping the item, the model fit improved: χ^2^(34, *N* = 501) = 111.08, *p* < 0.001, NFI = 0.948, CFI = 0.963, and RMSEA = 0.067 (90% confidence interval: 0.054, 0.081). Item loadings for the two specified factors were significant at *p* < 0.001, and ranged for each dimension as follows: Other-focused trust (0.667–0.853) and propensity to trust (0.480–0.763). Table 4 lists the items and their estimated standardized factor loadings. As expected, the two latent constructs were significantly correlated with one another (*r* = 0.65, *p* < 0.001).

#### Internal Consistency

Internal consistency for each of the scales was examined using Cronbach’s alpha. Both subscales demonstrated good reliability, with *a* = 0.89 for the other-focused trust sub-scale (5 items) and *a* = 0.75 for the propensity to trust sub-scale (5 items). The average inter-item correlations were *r* = 0.61 for other-focused trust and *r* = 0.39 for propensity to trust.

#### Associations With Criterion Measures

To examine convergent and discriminant validity, correlations between the two components of interpersonal trust and the individual difference variables were computed. Both other-focused trust and propensity to trust were positively correlated with self-esteem (*r* = 0.32, *p* < 0.001; *r* = 0.31, *p* < 0.001), social capital (*r* = 0.34, *p* < 0.001; *r* = 0.34, *p* < 0.001), and social support (*r* = 0.53, *p* < 0.001; *r* = 0.53, *p* < 0.001). Other-focused trust was positively correlated with propensity to trust (*r* = 0.54, *p* < 0.001). We compared the correlations between the two types of trust and the criterion measures, yet no significant differences were apparent in these analyses.

### Discussion

CFA demonstrated that the correlated two-factor model showed the best fit to the data. The result confirmed that interpersonal trust can be assessed in two components: Other-focused trust and propensity to trust. In this study, we did not find that each trust component uniquely correlated with each of the criterion measures. As in Study 1, personal self-esteem was associated with both components of trust in similar ways, and new to Study 2, the same was found for social support and social capital.

## Study 3: Criterion Validity

In this study, we tried to identify further variables that might be differentially associated with the subscales, to add evidence for their discriminant validity. Considering beliefs in a just world might be based on individuals’ opinions of others (Bègue, 2002; Otto and Dalbert, 2005), to extend our assessment of discriminant validity, we examined whether the two trust dimensions differentially predict general and personal beliefs in a just world. Beliefs in a just world are positively associated with optimism, mental health (self-esteem, satisfaction with life, and happiness), and perceptions of social justice (Correia and Vala, 2004). People who view the world as just (i.e., who have a general belief in a just world) are more likely to allow themselves to be trusting of others (Poulin and Cohen, 2008). On the other hand, people who have personally been treated fairly by others (i.e., who have a personal belief in a just world) are more likely to perceive others as trustworthy (Schindler and Reinhard, 2015). That is, though both beliefs in a just world are likely to be associated with both trust dimensions, we expected general beliefs in a just world to be more strongly associated with propensity to trust and personal beliefs in a just world to be more strongly associated with other-focused trust. We again measured satisfaction with life with the same scale as in Study 1.

### Materials and Methods

#### Participants

A total of 295 participants (age: 35.43 ± 11.09 years) were recruited through the Prolific Academic platform (see Table 1 for more details of the demographic information).

#### Measures

The 10-item other-focused and propensity to trust scale developed in Study 2 were again used to measure trust. Participants also completed the General (e.g., “I believe that, by and large, people get what they deserve”) and Personal Beliefs in a Just World Scales (e.g., “I am usually treated fairly”; Dalbert, 1999).

#### Procedure

The study was programmed using Qualtrics and distributed on the Prolific Academic platform. The survey protocol was approved by the University of Exeter’s Psychology Ethics Committee. After providing informed consent, participants responded to 10 items to assess interpersonal trust. Next, participants responded to the 5-item Satisfaction with Life Scale and the 13-item General and Personal Beliefs in a Just World Scale. All participants were compensated with £0.5, in line with this platform’s guidelines.

### Results

#### Internal Consistency

Internal consistency for each of the scales was examined using Cronbach’s alpha. Both subscales demonstrated good internal consistency, with *a* = 0.90 for the other-focused trust sub-scale (5 items) and *a* = 0.69 for the propensity to trust sub-scale (5 items). The average inter-item correlations were *r* = 0.63 for other-focused trust and *r* = 0.32 for propensity to trust.

#### Associations With Criterion Measures

The correlations between the two components and the individual difference variables are displayed in Table 6. Both components were positively correlated with satisfaction with life (*r* = 0.28, *p* < 0.001; *r* = 0.31, *p* < 0.001) and negatively associated with general and personal beliefs in a just world (*r* = –0.21, *p* < 0.001; *r* = –0.22, *p* < 0.001; *r* = –0.36, *p* < 0.001; *r* = –0.20, *p* < 0.001). Other-focused trust was positively correlated with propensity to trust (*r* = 0.53, *p* < 0.001). Again, we compared the magnitude of the correlations between the two types of trust and criterion measures (Table 6). The results showed that the correlation between other-focused trust and general beliefs in a just world was significantly greater compared to the correlation with propensity to trust.

**TABLE 6:** Comparison of correlation coefficients in Study 3.

	**Correlation coefficients**		***Z*-Score**	***p***
	**Other-focused trust**	**Propensity to trust**		
Satisfaction	0.283***	0.311***	–0.37	0.71
BJW-general	–0.361***	–0.201***	–2.11	**0.04**
BJW-personal	–0.206***	–0.218***	0.15	0.88

***p < 0.001. Bold values represent significant results.

### Discussion

By comparing the correlations between the two types of trust and other criterion measures, we found that other-focused trust was more strongly associated with general beliefs in a just world compared to propensity to trust. This suggests that other-focused trust, like general beliefs in a just world, may tap into people’s judgments of others in the social environment to a greater extent than propensity to trust (which may be more related to personality).

## General Discussion

This research presented findings from three studies conducted with samples from the United Kingdom. Psychometric analyses supported the feasibility of the proposed two-factor interpersonal trust model. The construct validity was confirmed by the evidence of item and factor dimensionality, as well as by evidence of the internal consistency of each dimension, indicating that the items reliably capture two distinct (though related) dimensions. Similarly, confirmatory factor analyses revealed that the two-factor model has a satisfactory-to-good fit to the data. The results also show that the two dimensions of trust have some shared and some unique relationships with other variables. Indeed, the divergences existed in the correlations between the two types of trust and some measures (extraversion, agreeableness, liking people, in Study 1 and general beliefs in a just world, in Study 3). Crucially, while propensity to trust was more strongly related to the personality variables and propensity to like other people, individuals who believed the world is generally a just place, we inclined to find others trustworthy. In sum, though the results showed that the two dimensions are closely related, they suggest that we successfully developed a two-component measure that assesses self- and other-focused trust.

The present studies have important theoretical and practical implications. First, we add to existing understandings of the dimensionality of interpersonal trust by distinguishing between self and other-focused trust. We underline that even though trust is always a relational construct, involving both self and others, to consider oneself trusting is not always the same as to trust others (see also Ashraf et al., 2006). Though previous research explored the relationships between other/self-profitable traits and trust (Wojciszke and Struzynska–Kujalowicz, 2007), existing evidence directly distinguishing between other-focused trust and propensity to trust incorporated willingness to incur costs into the conceptualization of propensity to trust (Yamagishi et al., 2015)—something that can be useful, but also has limitations. With this work, we introduce a new measure that taps into this distinction without explicitly considering willingness to incur costs. To be fair, behavioral demonstrations of trust might require a degree of both types of trust, but in different contexts different aspects of trust might be more or less relevant. Future research can now examine this. Second, these studies join others to suggest that to improve trust in interpersonal interactions one might need to attend to these two components of trust, one depending mainly on the individuals’ views of themselves as trusting and one depending on their views of others as worthy of trust. Trustworthiness (other-focused trust) is already considered an important factor when improving interpersonal trust (Heyns and Rothmann, 2015). Here, we highlight the importance of individuals’ propensity to trust, opening a new space for intervention.

Although we believe that our results offer important evidence that individual’s propensity to trust can be distinguished from their other-focused trust, we acknowledge there were limitations in the present research. On the one hand, all of the measures used were self-report absent any kind of narrative or framing in the cover story in our studies. It would be much helpful to show how the different dimensions of trust result in different attributions or behaviors in relation to others, providing more implications for application contexts. On the other hand, the fact that we only included United Kingdom-based participants in our sample means that care should be taken when using this measure in other countries. We also do not know to what extent the distinction we have made translates into other languages, or even cultures. It is, however, noteworthy that our samples were varied in terms of age range and education, offering greater validity than the more standard college-based samples.

Despite these limitations, now that we have developed this measure, future research might focus on whether these two trust components are driven by different factors, or differentially relate to behavior in social interactions. For example, other-focused trust is more likely to be driven by the identity of the other and associated beliefs, whereas propensity to trust might be more responsive to factors that affect one’s confidence in others more generally, irrespective of who they are, such as mood. In addition, (low) propensity to trust might be linked to socially inhibited behavior, whereas (low) other-focused trust might be associated with attempts to confront others, correct their untrustworthy attitude, or to seek others one might find more trustworthy. These different components of trust can also vary differently across time (Morselli and Glaeser, 2018). For example, as one gets to know that another person deserves one’s trust, other-focused trust might increase more than propensity to trust. At the same time, s propensity to trust might vary in a less linear fashion (increasing or decreasing), as it might depend on factors that fluctuate more often, such as mood.

## Conclusion

In conclusion, the present studies introduced a new measure of two important distinct dimensions of interpersonal trust: Other-focused trust and propensity to trust. Although these components are closely related, our findings showed that they can be conceptually and empirically distinguished. The nature of interpersonal interaction is a person interacting with another person or persons, and the interaction can be performed or not depends on both parties. Though there were series of studies focused on clarifying the construction of trust, the current studies focused on emphasizing both two parties and extending directional items that apart from each other. This work can thus be regarded as a step toward a more nuanced understanding of how trust is developed, how it varies, and how it plays out in social interactions.

## Data Availability Statement

The raw data supporting the conclusions of this article will be made available by the authors, without undue reservation.

## Ethics Statement

The studies involving human participants were reviewed and approved by the University of Exeter’s Psychology Ethics Committee. The ethics committee waived the requirement of written informed consent for participation.

## Author Contributions

The author confirms being the sole contributor of this work and has approved it for publication.

## Conflict of Interest

The author declares that the research was conducted in the absence of any commercial or financial relationships that could be construed as a potential conflict of interest.

## Publisher’s Note

All claims expressed in this article are solely those of the authors and do not necessarily represent those of their affiliated organizations, or those of the publisher, the editors and the reviewers. Any product that may be evaluated in this article, or claim that may be made by its manufacturer, is not guaranteed or endorsed by the publisher.

## Footnotes

1
https://www.danielsoper.com/statcalc/calculator.aspx?id=104

## Appendix

### Items in the Adjusted Social Capital Scale in Study 2

1. People around where I live are willing to help others.
2. Where I live, there is a close-knit, or “tight” relationship where people generally know one another.
3. If I had to borrow £30 in an emergency, I could borrow it from others where I live.
4. People where I live generally don’t get along with each other.
5. If I were sick I could count on others where I live to shop for groceries for me.
6. People where I live do not share the same values.

### Items in the Adjusted Brief Form of the Perceived Social Support Questionnaire in Study 2

1. Where I live, people give others a lot of understanding and security.
2. Where I live, people can easily find someone very close to them whose help they can always count on.
3. Where I live, people are happy to lend something to others who need it.
4. Where I live, people can find someone with whom they like to do things.
5. Where I live, people are happy to handle important things for others who are sick.
6. Where I live, people are happy to be turned to if others are very depressed.
