# Supplementary material for: Assessing Two Dimensions of Interpersonal Trust: Other-Focused Trust and Propensity to Trust
Source: Front Psychol. 2021 Jul 27;12:654735. doi: 10.3389/fpsyg.2021.654735 (PMC8353080; doi:10.3389/fpsyg.2021.654735)
Supplement: Supplementary file 1 [file Table_1.DOCX]

# **Supplementary Materials**

**Table 1 The effect of gender on trust scores in Study 1, Study 2, and Study 3**

|  | **Other-focused trust** | | | | **Propensity to trust** | | | |
| --- | --- | --- | --- | --- | --- | --- | --- | --- |
|  | **Male**  ***M (SD)*** | **Female**  ***M (SD)*** | ***t*** | ***p*** | **Male**  ***M (SD)*** | **Female**  ***M (SD)*** | ***t*** | ***p*** |
| **Study 1** | 3.64 (.79) | 3.64 (.76) | .03 | .978 | 3.68 (.71) | 3.81 (.73) | –1.94 | .053 |
| **Study 2** | 3.51 (.79) | 3.49 (.79) | .24 | .810 | 3.89 (.70) | 3.91 (.70) | –.38 | .701 |
| **Study 3** | 3.43 (.79) | 3.47 (.82) | –.36 | .721 | 3.85 (.52) | 3.85 (.71) | .02 | .985 |

**Table 2 The effect of education on trust scores in Study 1, Study 2, and Study 3**

|  | **Other-focused trust** | | | | **Propensity to trust** | | | |
| --- | --- | --- | --- | --- | --- | --- | --- | --- |
|  | **High school**  ***M (SD)*** | **BSC or above**  ***M (SD)*** | ***t*** | ***p*** | **High school**  ***M (SD)*** | **BSC or above**  ***M (SD)*** | ***t*** | ***p*** |
| **Study 1** | 3.49 (.79) | 3.72 (.75) | –3.09 | .002 | 3.75 (.73) | 3.78 (.71) | –.40 | .689 |
| **Study 2** | 3.40 (.78) | 3.60 (.79) | –2.73 | .007 | 3.93 (.69) | 3.88 (.71) | .89 | .373 |
| **Study 3** | 3.36 (.83) | 3.55 (.76) | –2.01 | .045 | 3.85 (.67) | 3.84 (.67) | .05 | .959 |
